# A Multiphase Flow in the Antroduodenal Portion of the Gastrointestinal Tract: A Mathematical Model

**DOI:** 10.1155/2016/5164029

**Published:** 2016-06-19

**Authors:** P. V. Trusov, N. V. Zaitseva, M. R. Kamaltdinov

**Affiliations:** ^1^Federal Scientific Center For Medical and Preventive Health Risk Management Technologies, 82 Monastyrskaya Street, Perm 614000, Russia; ^2^Department of Mathematical Simulation of Systems and Processes, Perm National Research Polytechnic University, 29 Komsomolsky Avenue, Perm 614000, Russia

## Abstract

A group of authors has developed a multilevel mathematical model that focuses on functional disorders in a human body associated with various chemical, physical, social, and other factors. At this point, the researchers have come up with structure, basic definitions and concepts of a mathematical model at the “macrolevel” that allow describing processes in a human body as a whole. Currently we are working at the “mesolevel” of organs and systems. Due to complexity of the tasks, this paper deals with only one meso-fragment of a digestive system model. It describes some aspects related to modeling multiphase flow in the antroduodenal portion of the gastrointestinal tract. Biochemical reactions, dissolution of food particles, and motor, secretory, and absorbing functions of the tract are taken into consideration. The paper outlines some results concerning influence of secretory function disorders on food dissolution rate and tract contents acidity. The effect which food density has on inflow of food masses from a stomach to a bowel is analyzed. We assume that the future development of the model will include digestive enzymes and related reactions of lipolysis, proteolysis, and carbohydrates breakdown.

## 1. Introduction

A human body is constantly interacting with the environment: it consumes vital nutrients and is impacted by chemicals found in food and drinking water [1–3]. In addition to irritating the walls of the digestive tract, the chemicals can accumulate in a body and have a systemic effect as they are found in blood flowing to the organs. Moreover, irregular and imbalanced nutrition may result in various disorders in organs and systems [4–6]. Statistical approaches to assessing the incidence and death rates associated with the environmental factors are primarily based on developing “dose-response” relationships. Such approaches do not directly deal with variable exposure of factors, as well as exposure continuance and its mechanisms. Physiological approaches can give deeper insight into the processes related to the development of disorders in organs and systems. Over the last few decades, there have been numerous research papers on the mathematical models representing cardiovascular, respiratory, digestive, and other systems. Organs and systems are interconnected throughout a human body; thus, it is advisable to take these relationships into account when developing mathematical models.

The group of researchers, including the authors of this article, is working on a multilevel mathematical model of damage accumulation in a human body associated with environmental exposure (chemical, physical, social, etc., factors) [7, 8]. At the “macro-level”, we are analyzing the interaction between organs and systems with the help of ordinary differential systems that describe the evolution of damage. The level of damage is assigned a numerical value between zero and one. The value is zero when there are no functional disorders in an organ; one when the functions fail completely. Several mechanisms are considered: natural (ageing and self-restoration), non-normative environmental impact, medical treatment, and preventive measures. It is clear that this model gives only a rough assessment of the bodily damage and requires a larger amount of empirical data to identify the model. For a more detailed description of the interactions between systems and organs and the determination of causal relationships, it is necessary to consider the next level (“mesolevel”) for models of the cardiovascular, respiratory [9], digestive, neuroendocrine [10], and other systems. In the future, it might be necessary to develop the “microlevel” models to describe the cellular level.

Due to broadness of the research tasks, this article focuses on only one fragment related to the digestive system modeling at the “mesolevel.”

*The purpose of the research* is to build a mathematical model for the multiphase flow in the antroduodenal portion of the gastrointestinal tract taking chemical absorption, acid and alkaline secretion, and the tract walls motion into account.

Experimental approaches are traditionally used to investigate the digestive tract. They help in assessing the functional, morphological, and geometrical parameters of organs. An MRI scan can quickly produce detailed images of body internals in different cross-sections. It can help monitoring the motor activity of the tract and the gastric emptying rate [11, 12]. A disadvantage of the method is that the equipment is expensive. Moreover, the test is conducted when a person is in a lying position, but the food distribution process in the tract is different than in a sitting or standing position [13].

Also, scintigraphy helps visualize the process of digestion. It is a form of diagnostic testing where radioisotopes are taken internally and the emitted radiation is captured by external detectors. This testing can be used to analyze the distribution of food particles in the gastrointestinal tract over time and assess the rate of gastric emptying [14]. The main disadvantage of scintigraphy is the use of radioactive substances, which may cause negative health effects. Ultrasound produces images of the geometrical shapes of organs and helps assess the motion activity of the GI tract [15, 16]. The experimental methods of electrogastrography and magnetogastrography are used to assess the motor function by registering the characteristics of electromagnetic fields around the organs of the digestive tract [17]. Image processing methods, such as spatiotemporal motility mapping for smooth muscle activity in ex vivo or in vivo conditions [18] and electrophysiology [19], have also given numerous insights into our understanding of the gastrointestinal motility. The above methods except for intracavitary ultrasound do not involve penetration of the body with sensors or devices which is a definite advantage.

To measure the local intracavitary pressure in various portions of the digestive tract, it is necessary to use methods that involve introducing a foreign object into the body, for example, sensors, catheters, or vessels [20]. It should be noted that the precision of measurements is lacking, and there are no alternative approaches to measure the pressure inside the GI tract. To assess the secretory function of the stomach, it is necessary to use a pH meter which is a scientific instrument that measures acidity in the GI tract [21]. This procedure can be combined with endoscopy, which is a nonsurgical procedure used to examine a person's digestive tract with the help of an optical tube passed through the mouth. It should be noted that introduction of a device inside a body can skew the test results. Most experimental methods are aimed at studying the body at rest, which does not always correspond to real conditions.

In vitro studies can help save time and money, as opposed to in vivo studies. One of the most common ways to imitate GI processes is through sequential adding of substances and enzymes into the test tube with food [22]. This method is commonly used to determine the rate of decomposition of medical preparations and food particles into digestible elements [23]. More comprehensive machines have several cameras with computer-controlled secretion and peristaltic motility [24]. The biggest disadvantage of using in vitro tests is the necessity to create a realistically shaped GI tract with moving walls.

It should be noted that experiments are not restricted to the measurement of physiological variables but also to flow/digestive variables, as in the application of residence time distribution methods to quantify mixing in the small intestine [25, 26] and biochemical variables [27].

Other major disadvantages of the experimental approaches include significant financial and time expenditures and difficulties measuring some of the indicators associated with penetrating the human body. The inability to expose subjects to hazardous substances is also a limiting factor. Advancements in experimental equipment and medical-image processing have improved scientific studies on mathematical modeling of digestive processes in the last several decades. Mathematical modeling can help decrease financial expenditures associated with a physical experiment, include/exclude individual factors or combination thereof, and also estimate quantitative parameters of the process. At the preparation stage, however, it is necessary to use experimental data for the identification and verification of the model parameters.

Today there are only a few models that study the digestive system as a whole. As a rule, researchers focus on building models that describe specific processes in organs and their portions, for example, in the oral cavity [28–30], esophagus [31–33], stomach [34–37], and intestine [38, 39]. The models that describe multiphase flows in the stomach and intestine are most interesting due to the nonstandard geometry and multiplicity of effects. Available models mainly account for the motor function of the GI tract which involves the spread of peristaltic waves.

Biochemical reactions, secretion of digestive glands, and food dissolution are given less consideration. To study the evolution of the stomach geometric shape, the MRI results are generally used. For example, one of the first studies on mathematical modeling of gastric digestion simulated the wave propagation in the antrum that promotes mixing of the food [40, 41]. A series of studies on the modeling of flows in a closed stomach presents an algorithm on building a simplified 3D gastric shape [42], also, it includes a computational study of the impact that fluid viscosity [43], density, and volume fraction of the second phase particles [44] exert on the flow characteristics. The results of the computational 3D modeling using an anatomically realistic shape of the stomach indicate that when the stomach is partially full, a body posture affects the mixing of the food [45]. Also, the results of 2D modeling describe some interesting aspects including transfer of the food through the pyloric sphincter [46], distribution of pepsin in the antrum [47], and two-phase flow in the stomach including evacuation into the bowel [48]. The models of intestine digestion [49, 50] are normally limited by 1D and 2D cases, but they offer a rich content because they cover biochemical reactions [51], elasticity of the tract walls, and absorption of the substances into the blood stream [52]. Digestive processes are complicated, so it might be necessary to study them at different levels. Flow in the gut is “multiscale”; for example, the small intestine villi generate flow at a microscale (<0.001 m) that can interact with the flow at the organ scale (≈0.01 m) [38, 53].

This demonstrates that the models describing digestive processes need improvement (i.e., development of the approaches describing multiphase flows in a 3D case). At the same time, it is necessary to consider the biochemical reactions, digestive gland secretion, and regulatory mechanisms as well as functional tract disorders and interrelations with other organs and systems.

It is possible to use multicompartment kinetic models to take a closer look at the entry of substances into the circulatory system [54]. In fact, it is one of the ways in which organs and systems interact at the “macrolevel.” Also, given the peculiarities of digestion in various portions of the tract, it is possible to use simplified models based on the theory of chemical reactors [55]. However, simplified approaches make it difficult to capture individual characteristics of a patient, especially when it comes to the geometric shape of the tract and peristaltic parameters. In the attempts to locate a disorder, researchers suggest using patient-oriented approaches based on the results of real tests (e.g., ultrasonography and magnetic resonance imaging).

At the earlier stages of developing a multilevel approach, we outlined the concept of the digestive system modeling at the “mesolevel” including digestion submodels in the oral cavity, stomach, and the gut [56]. Also, we generated an algorithm for restoring a simplified 3D shape of the antroduodenal portion of the tract using the ultrasound results. Throughout the studies, we obtained some results characterizing the process of a one-phase flow bearing the spread of peristaltic waves in the antrum, opening/closing of the gastric outlet, and functional motor disorders [57] in mind.

## 2. Conceptual Statement

**Figure 1:**
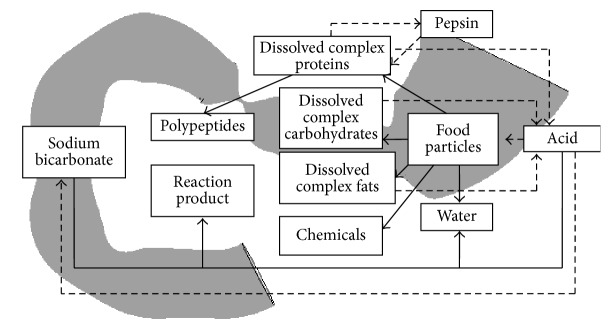
A simplified model of digestion in the antroduodenal portion of the digestive tract (a solid line indicates mass transition and a dotted line feedback and managing impact).

This paper focuses on multiphase environment. The first phase is a multicomponent fluid with components dissolved at the molecular level: water, hydrochloric acid, sodium bicarbonate, the reaction product with hydrochloric acid and sodium bicarbonate (carbon dioxide and sodium chloride), dissolved proteins, fats, carbohydrates, chemicals, polypeptides, and pepsin (Figure 1).

Food particles of different sizes are viewed as separate phases (in the general case-*n* − 1 phase). Hydrochloric acid dissolves the food particles, and the mass transforms into the first phase components (dissolved proteins, fats, carbohydrates, chemicals, and water); some of the particles are transferred into a phase of a smaller size. Dissolution of food particles (reduction in size) may take place until a certain limit set for this type of food. This means that the particles of the minimum size may not be dissolved. The rate of mass exchange is linearly dependent on the acidity level (pH). In the neutral or alkaline environment, the mass exchange equals zero. At a first approximation, the food particles are described by a model of fluid with various viscosity; we put forward the hypothesis that the pressure in different phases is the same. The forces of interphase interaction are thought to be proportional to the difference in velocities of the interacting phases.

The model includes the neutralization of the acid with sodium hydrogen carbonate

(1) $$\begin{array}{c} NaHCO_{3}^{}+HCl⟶\\ NaCl+H_{2}CO_{3}⟶\\ NaCl+H_{2}O+CO_{2} \end{array}$$

The mixture of sodium chloride and carbon dioxide (carbonic acid) is regarded as one component. Acid neutralization that takes place when contacting with food (hydrolysis of peptide bonds in the cell structure) is disregarded. An enzymatic reaction between pepsin and protein transforms complex proteins into polypeptides.

The model takes three main functions of the digestive tract, namely, motor, secretory, and absorption, into account.

The acid secretion rate depends on the concentration of dissolved proteins, fats, and carbohydrates along the tract wall (in the region of acid secretion). The pepsin secretion rate depends on the concentration of dissolved proteins along the tract wall (in the region of pepsin secretion). The sodium bicarbonate secretion rate depends on the average acid concentration near the stomach walls (feedback). Any damage to the digestive tract and the pancreas reduces the secretion rate. The motor function in the antroduodenal portion includes periodic contraction/relaxation of the tract wall muscles. The spread of peristaltic waves in the antrum, motility of the gastric outlet, and peristaltic waves in the duodenum are also taken into account. Any damage in the tract reduces the amplitude of the wave. The amount of chemicals absorbed into blood is proportional to the difference between concentration at the border of the tract cavity and the blood concentrations.

Temperature impacts digestion, namely, the biochemical reactions rate, the food dissolution, and the physical properties of the digestive fluid (viscosity and density). In most cases, food enters the stomach in small amounts, thus making the gastric contents reach body temperature rather rapidly; temperature impact seems to be important only in the initial digestion stages [65]. This circumstance can be crucial only for some foods which do not stay in the stomach for long. This includes fluid which contains little nutrients [63, 66–71]. The current study presents an analysis of the digestion process at the stage when the food temperature in the stomach has already reached body temperature (37°С). For this reason, the temperature effects are not reflected in all equations.

**Figure 2:**
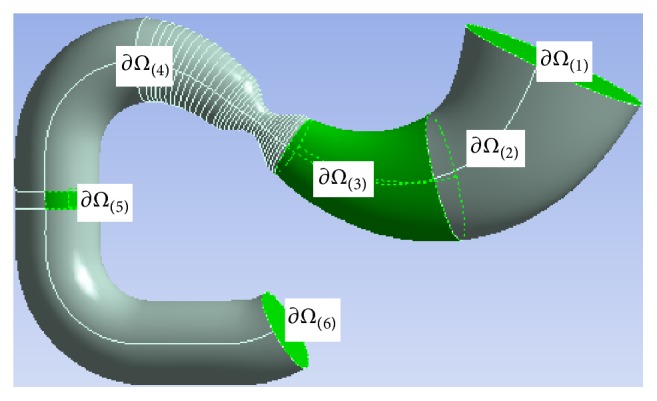
Identifying subareas by functional and geometric features, ∂*Ω*_(2)_ is the acid secretion area, ∂*Ω*_(3)_, ∂*Ω*_(4)_, and ∂*Ω*_(5)_ are the alkaline secretion area, and ∂*Ω*_(1)_, ∂*Ω*_(6)_ are the inlet/outlet section.

The results below were obtained when analyzing a typical (in terms of size and shape) antroduodenal portion of the digestive tract. This simplification takes place at the stage of the model adjustment; further implementation of the model will require personal data. The reconstruction algorithm of the 3D modeling area is based on approximation of a real geometrical shape with the finite number of ellipses covered with a straight membrane. The ellipses change in size and are set on a normal plane to the “center line” of the antroduodenal portion. It seems practical to divide the boundary of the modeling area into several parts, based on the functional and geometrical characteristics, for which we will introduce a damaged organ area (Figure 2).

We have identified four boundaries. Acid secretion takes place in the upper part of the stomach ∂*Ω*_(2)_. Sodium hydrogen carbonate is secreted in the lower part of the stomach ∂*Ω*_(3)_, adjacent to the gastric outlet. Sodium hydrogen carbonate is also secreted in the area of the gastric outlet and duodenum ∂*Ω*_(4)_. In the central part of the duodenum, we have outlined an area ∂*Ω*_(5)_, which imitates the inflow of the pancreatic juice. The pancreatic juice also contains sodium bicarbonate. Areas ∂*Ω*_(1)_ and ∂*Ω*_(6)_ have open boundaries (the inlet-outlet section). Each section of the tract wall ∂*Ω*_(*l*)_ has its own damage *D*_(*l*)(*m*)_, $l=\bar{2,5}$, determined by a respective evolutionary equation. Functionality of the tract wall section is related to the damage with the following formula: *F*_(*l*)(*m*)_ = 1 − *D*_(*l*)(*m*)_, functionality that is different from 1, is shown by a quantitative reduction of the secretory *F*_(*l*)(1)_, absorption *F*_(*l*)(2)_, and motor *F*_(*l*)(3)_ tract functions.

## 3. Mass and Momentum-Conservation Equations

Generally advection-diffusion equations for the first-phase components can be given as

(2) ∂∂tρ1α1Yi+∇·ρ1α1v1Yi=−∇·Ji+Ri+Si+∑jmji′,r∈Ω¯,  t∈0;T,  i=0,I¯,  j=2,n¯,

where *i* is a component index of the first phase: hydrochloride acid (*i* = 0), reaction product of hydrochloride acid and sodium hydrogen carbonate (carbon dioxide and sodium chloride) (*i* = 1), sodium hydrogen carbonate (*i* = 2), solute proteins (*i* = 3), fats (*i* = 4), carbohydrates (*i* = 5), chemicals (*i* = 6), pepsin (*i* = 7), polypeptides (*i* = 8), and water (*i* = 9); *j* is a phase index, index *j* = 1 corresponds to the first phase which is a multicomponent liquid, and index *j* > 1 corresponds to phases of food particles with different sizes, which in first approximation are described with a model of liquid with different viscosity; *ρ*_(*j*)_ is a density of the *j*-phase, kg/m^3^; *α*_(*j*)_ is a volume fraction of the *j*-phase; *Y*_(*i*)_ is a mass fraction of the *i*-component of the first phase; ∇ is a nabla operator, ∇ = (∂/∂*x* + ∂/∂*y* + ∂/∂*z*), so we use Cartesian coordinate system; ∇· is a divergence operator; for example, ∇·**v** = (∂*v*_*x*_/∂*x* + ∂*v*_*y*_/∂*y* + ∂*v*_*z*_/∂*z*); **r** is a radius-vector of spatial points, m; in Cartesian coordinate system **r** = *x* **e**_*x*_ + *y* **e**_*y*_ + *z* **e**_*z*_ = {**x**, **y**, **z**}; **v**_(*j*)_ is a vector of *j*-phase velocity, m/s; **J**_(*i*)_ is an intensity vector of mass flow of the first phase *i*-component due to diffusion processes, kg/(m^2^·s); *R*_(*i*)_ is an intensity of mass sources of the first-phase *i*-component due to reactions between components, kg/(m^3^·s); *S*_(*i*)_ is a mass sources intensity of the *i*-component, kg/(m^3^·s); *m*_(*j*)(*i*)_′ is an augend in mass balance equation, determining the transition intensity from *j*-phase into the first phase *i*-component, kg/(m^3^·s); *t* is an independent variable (time), s; *Ω* is an interior of the whole area; ∂*Ω* is the area boundary; $\bar{\Omega }=\Omega \cup \partial \Omega$ is the area closure (area interior and its boundary); ∂*Ω*_(*l*)_ is *l*-boundary of area, $l=\bar{1,L}$; we introduce secretion as mass sources in layer, adjacent to the tract wall, so we define *Ω*_(*l*)_ as internal layer, adjacent to the *l*-boundary, $l=\bar{1,L}$.

The mass conservation equation for the liquid phases of the food particles in general terms can be expressed as

(3) ∂∂tρjαj+∇·ρjαjvj=−mji′+mj−1j′′−mjj+1′′,r∈Ω¯,  t∈0;T,  i=0,I¯,  j=2,n¯,  m12′′=mnn+1′′=0,

where *m*_(*j*−1)(*j*)_′′ is an augend in mass balance equation, determining the transition intensity from (*j* − 1)-phase into *j*-phase, kg/(m^3^·s).

The system of mass conservation equation is complemented by the following 2 equations:

(4) ∑iYi=1,∑jαj=1,i=0,I¯,  j=1,n¯.

The momentum-conservation equation can be written as

(5) ∂∂tα1ρ1v1+∇·α1ρ1v1v1=−α1∇p+∇·τ1+α1ρ1g+∑jK1jv1−vj+∑j∑i=3,4,5,6,9mji′vj,∂∂tαjρjvj+∇·αjρjvjvj=−αj∇p+∇·τj+αjρjg+∑qKjqvj−vq−∑i=3,4,5,6,9mji′vj+mj−1j′′vj−1−mjj+1′′vj,r∈Ω,  t∈0;T,  j=2,n¯,  q=2,n¯,  m12′′=mnn+1′′=0,τj=αjηj∇vj+∇vjT,j=1,n¯,  r∈Ω¯,

where *p* is phases mixture pressure, Pa; **τ**_(*j*)_ is a stress tensor of the *j*-phase, Pa; **g** is a vector, characterizing mass force impact, m/s^2^; *K*_(*j*)(*q*)_ is a coefficient of interphase interaction *j* and *q*-phase, kg/(m^3^·s); *η*_(*j*)_ is a shear viscosity of the *j*-phase, Pa·s.

## 4. Diffusion Term

Since we do not consider the temperature effects, the diffusion is determined only by the concentration gradient. For the laminar flow, the intensity vector of the *i*-component mass flow due to diffusion processes can be presented as (Fick's law)

(6) $$\begin{array}{c} J_{\left(i\right)}=-\rho_{\left(1\right)}K_{\left(i\right)}\nabla Y_{\left(i\right)}, \end{array}$$

where *K*_(*i*)_ is a diffusion coefficient of the first-phase *i*-component in digestive tract cavity, m^2^/s. At a first approximation, *K*_(*i*)_ is thought to be equal for all components. During the peristaltic activity, the mass is transferred mainly due to advection rather than diffusion. However, when the motor function of the GI tract is damaged, diffusion can become more significant. For this reason, it is impractical to exclude diffusion (6) from (2).

## 5. Reaction Mass Sources

The summand of the mass source due to the neutralization reaction is proportional to the product of the hydrochloric acid and sodium hydrogen carbonate concentration:

(7) $$\begin{array}{c} R_{\left(i\right)}=k_{\left(0\right)\left(2\right)}M_{\left(i\right)}C_{\left(0\right)}C_{\left(2\right)},\;i=0,1,2,9, \end{array}$$

where *k*_(0)(2)_ is the reaction rate constant, m^3^/(s·kmol), *M*_(*i*)_ is the molar mass of the first-phase *i*-component, *R*_(*i*)_ is taken as negative for the reagents, and positive for the reaction products. Molar concentration of the *i*-component can be calculated with the help of the following ratio:

(8) $$\begin{array}{c} C_{\left(i\right)}=\frac{\alpha_{\left(1\right)}Y_{\left(i\right)}\rho_{\left(1\right)}}{M_{\left(i\right)}}. \end{array}$$

The summand of the mass source due to enzymatic reaction between proteins and pepsin looks as follows:

(9) Ri=k37f37pHα12ρ12Y3Y7/M7k37′+α1ρ1Y3/M3,i=3,8,

where *k*_(3)(7)_, *k*_(3)(7)_′ are reactions rates constants, 1/s, kmol/m^3^; when *i* = 3  *R*_(*i*)_ is taken as negative, when *i* = 8  *R*_(*i*)_ is taken as positive. Formula (9) is a classic Michaelis-Menten ratio [72] that describes the enzymatic reaction rate modified by the dependency *f*_(3)(7)_(pH) on the acidity level. In the classic formula, the effect of external conditions (temperature, acidity, etc.) is expressed by the coefficient *k*_(3)(7)_. The modified version of formula (9) is based on the assumption that it is possible to divide the coefficient into two components in order to express the more important factor which is acidity. The rate of this reaction in accordance with (9) is increasing linearly as the mass fraction of enzyme *Y*_(7)_ grows. The rate of the reaction is growing nonlinearly as the mass fraction of the dissolved proteins *Y*_(3)_ increases up to a certain limit defined by a constant *k*_(3)(7)_′.

Following the results of the experimental studies [62], we can suggest using a quadratic function for *f*_(3)(7)_(pH) = *a*_(3)(7)_ · pH^2^ + *b*_(3)(7)_ · pH + *c*_(3)(7)_ at a first approximation. The maximum reaction rate for most proteins is reached at pH = 2 (vertex of the parabola, the optimal level of pepsin action).

## 6. Secretion Mass Sources

We introduce secretion as mass sources in layer, adjacent to the tract wall, so the mass source due to secretion is presented as

(10) Si=Fl1Sil,r∈Ωl,  l=2,5¯,  ∑l .

*S*_(*i*)(*l*)_ is an intensity of mass sources of the *i*-component of the first phase in area *Ω*_(*l*)_, kg/(m^3^·s); *F*_(*l*)(*m*)_ is functionality of the *l*-sub-area, characterizing *m*-function (*m* = 1 means secretion function); symbol ∑l  means that index *l* is not summation index. Thus, the secretory flow is proportional to functionality; under *F*_(*l*)(1)_ = 0 no secretion takes place; when *F*_(*l*)(1)_ = 1 the source intensity corresponds to secretion with no functional disorders *S*_(*i*)_ = *S*_(*i*)(*l*)_. Strictly speaking, acid secretion depends on several factors [73]. But it is possible to distinguish a primary link (food); it starts the interactions chain that regulates acid secretion. The volume and nature of secretion over time are determined by the type of consumed food [58]. The food content is determined by receptors located on the tract walls. For this reason, the acid secretion rate depends on mass concentrations of the food components dissolved in close proximity to the tract wall and can be described by the ratio:

(11) S02=s020+∑is02iρ1α1Yi2s02′+∑iρ1α1Yi2,i=3,4,5,

where [*ρ*_(1)_ *α*_(1)_ *Y*_(*i*)_]_(*l*)_ is a mean mass concentration of the *i*-component of the first phase in area *Ω*_(*l*)_, kg/m^3^; *s*_(*i*)(*l*)_^0^, *s*_(*i*)(*l*)_′ are secretion rates constants of the first-phase *i*-component in area *Ω*_(*l*)_, kg/(m^3^·s), kg/m^3^; *s*_(0)(2)(*i*)_ defines acid secretion rate depending on the *i*-component concentration, kg/(m^3^·s); *s*_(*i*)(*l*)_^0^ corresponds to basal secretion level; *s*_(*i*)(*l*)_′ is the limiting constant. Ratio (11) is an analogue of the Michaelis-Menten equation and is used to describe the effects that include saturation. In other words, there is a maximum value for secretion intensity. When this value is reached, further densification of dissolved food components will not increase secretion.

The rate of pepsin secretion is described by a ratio similar to (11) but it depends solely on the mass concentration of dissolved proteins:

(12) $$\begin{array}{c} S_{\left(7\right)\left(2\right)}=s_{\left(7\right)\left(2\right)}^{0}+\frac{s_{\left(7\right)\left(2\right)}{\left[\rho_{\left(1\right)}\alpha_{\left(1\right)}Y_{\left(3\right)}\right]}_{\left(2\right)}}{s_{\left(7\right)\left(2\right)}^{\prime }+{\left[\rho_{\left(1\right)}\alpha_{\left(1\right)}Y_{\left(3\right)}\right]}_{\left(2\right)}}. \end{array}$$

The rate of sodium bicarbonate secretion is described by a similar rate and depends on acid concentration:

(13) $$\begin{array}{c} S_{\left(2\right)\left(l\right)}=s_{\left(2\right)\left(l\right)}^{0}+\frac{s_{\left(2\right)\left(l\right)}{\left[\rho_{\left(1\right)}\alpha_{\left(1\right)}Y_{\left(0\right)}\right]}_{\left(2\right)}}{s_{\left(2\right)\left(l\right)}^{\prime }+{\left[\rho_{\left(1\right)}\alpha_{\left(1\right)}Y_{\left(0\right)}\right]}_{\left(2\right)}},\;l=3,4,5. \end{array}$$

Ratio (13) reflects a well-known experimental fact [58] that the enhancement of acid secretion in the area *Ω*_(2)_ results in increased hydrogen carbonate secretion in the areas *Ω*_(3)_, *Ω*_(4)_, and *Ω*_(5)_ as it is needed to neutralize the acid. The water secretion rate in the upper part of the stomach *Ω*_(2)_ is proportional to the rate of acid secretion *S*_(9)(2)_ = *s*_(9)(2)_ *S*_(0)(2)_. The water secretion rate in the area of *Ω*_(3)_, *Ω*_(4)_, *Ω*_(5)_ is proportional to the rate of alkaline secretion *S*_(2)(*l*)_. Therefore, we assume that the acid and alkaline concentrations in the gland secreted fluids are constant at a first approximation.

## 7. Absorption Mass Sources

Chemicals are absorbed from GI tract through the walls by diffusion. The intensity of chemical component mass source can be written as

(14) Si−Fl2Sil=−Fl2silρ1α1Yil−hiCib,i=6,  l=2,3,4,5,

where *h*_(*i*)_ is a nondimensional coefficient that shows critical concentration ratio of the *i*-substance in tract cavity and blood. When it is reached, diffusion process into blood starts; *C*_(*i*)_^*b*^ is mass concentration of the *i*-chemical in blood, kg/m^3^. The mass source (14) is linearly dependent on functionality; under *F*_(*l*)(2)_ = 1, the absorption rate corresponds to absorption with no functional disorders. Under *F*_(*l*)(2)_ = 0, no absorption takes place. The general equation can be written for any quantity of chemical components at this stage. We review only one component *i* = 6 to exemplify the above.

## 8. Interphase Exchange

The summand responsible for the mass transition from *j*-phase into the first-phase *i*-component, for the sphere-shaped particles, can be written as

(15) $$\begin{array}{c} m_{\left(j\right)\left(i\right)}^{\prime }=\beta_{\left(i\right)}\frac{6k\cdot \alpha_{\left(j\right)}}{d_{\left(j\right)}}, \end{array}$$

where *k* is mass-transfer coefficient from a particle into the first phase, kg/(m^2^·s) (mass transition speed from the area unit); *d*_(*j*)_ is the particle diameter of the *j*-phase; the rate of mass transition in the particle dissolution is proportional to the fraction of components in the particle *β*_(*i*)_. Food particles consist of proteins, fats, carbohydrates, chemicals, and water, respectively, *i* = 3,4, 5,6, 9.

At this stage, the coefficient *k* = *k*_phys_ · *f*(pH) = *k*_phys_ · *a* · pH^*b*^ is given by exponential function of pH [64], where *k*_phys_ is a mass-transfer coefficient from a particle into the first phase at physiologically normal acid level in the stomach body, kg/(m^2^·s). Under normal functioning, the gastric environment has an optimal level of acidity for dissolution of food particles (approximately at pH = 1.8, *f*(pH) = 1); under acidity reducing the dissolution rate goes down.

The coefficient of the interphase interaction between the *j*-phase and the *q*-phase for the sphere-shaped particles under Re_(*j*)(*q*)_ ≤ 1000 (liquid phases move at a low velocity) can be presented as follows [74]:

(16) Kjq=Kqj=72αjαqηjdj+dq21+0.15 Rejq0.687,j=2,n¯,  q=2,n¯,Kj1=K1j=18αjα1η1dj21+0.15 Rej10.687,j=2,n¯,

where Re_(*j*)(*q*)_ = Re_(*q*)(*j*)_ = 0.5 · *ρ*_(*j*)_|**v**_(*j*)_ − **v**_(*q*)_|(*d*_(*j*)_ + *d*_(*q*)_)/*η*_(*j*)_, viscosity and density of particles phases ($j=\bar{2,n}$) are assumed equal, and Re_(*j*)(1)_ = Re_(1)(*j*)_ = *ρ*_(1)_|**v**_(*j*)_ − **v**_(1)_|*d*_(*j*)_/*η*_(1)_.

The summand responsible for the mass transition from *j* − 1 phase to *j*-phase for the sphere-shaped particles can be presented as follows:

(17) $$\begin{array}{c} m_{\left(j\right)\left(j+1\right)}^{\prime \prime }=\frac{6d_{\left(j+1\right)}^{3}k\alpha_{\left(j\right)}}{d_{\left(j\right)}\left(d_{\left(j\right)}^{3}-d_{\left(j+1\right)}^{3}\right)}. \end{array}$$

## 9. Boundary and Initial Conditions

Given the peristaltic motion, the following kinematic boundary conditions (no-slip conditions) are set on the tract walls:

(18) vjt,r=drtdt,j=1,n¯,  t∈0;T,  rt∈∂Ωl,  l=2,3,4,5,

where **r**(*t*)∈∂*Ω*_(*l*)_ is a radius-vector of wall material point in the antroduodenal portion of the digestive tract. The contractions amplitude is proportional to the functionality of the area *F*_(*l*)(3)_. At other boundaries (inlet/outlet section), a zero-pressure gradient is set:

(19) $$\begin{array}{c} \nabla p=0,\;t\in \left[0;T\right),\;\;r\left(t\right)\in \partial \Omega_{\left(l\right)},\;\;l=1,6. \end{array}$$

The initial conditions look as follows:

(20) αjt,r=αj0,Yit,r=Yi0,vjt,r=vj0,r∈Ω¯,  t=0.

## 10. Identification of Model Parameters

Parameters identification is one of the most challenging parts of mathematical model development especially in biomedical studies. The hardest part is to identify spatially distributed flow characteristics of the gastric content at different digestion stages. At current modeling stage, we have been able to identify most of the parameters based on the literary data (Table 1). Basic motor parameters of a wave in the antrum (in the absence of functional disorders) are set as described in the available literature [40] and ultrasound results [56]. Periodicity is 18 s, wave width is 0.02 m, and amplitude is 0.009 m. A wave is generated in the stomach and spreads to the gastric outlet at 0.0022 m/s for 38 s. Parameters of a wave in the duodenum are as follows: periodicity is 9 s, wave width is 0.04 m, and amplitude is 0.0035 m. The wave is generated in close proximity to the gastric outlet and spreads at 0.005 for 36 s. The gastric outlet opens and closes for 2 s every 18 s. As a result, under the set parameter, the motor activity of the antrum, gastric outlet, and duodenum is synchronized. The geometry of the computational region changes periodically.

Parameters for the component secretion were based upon the data on maximal and basal secretion rate depending on the type of consumed food (proteins, fats, and carbohydrates) [58]. Maximal HCl secretion in the stomach 20 · 10^−3^ mol/h = 0.73 g/h = 2.02 · 10^−7^ kg/s amounts to approximately 0.58% of solution mass fraction (hence, *s*_(9)(2)_ = 171.3), and, respectively, water flow in the stomach amounts to 346 · 10^−7^ kg/s. The basal level of hydrogen chloride secretion is approximately 5 times lower than the maximal level *s*_(0)(2)_^0^ = 0.404 · 10^−7^/*V*_(2)_ kg/(m^3^·s). Maximal secretion corresponds to proteins: that is why *s*_(0)(2)(3)_ = 1.616 · 10^−7^/*V*_(2)_ kg/(m^3^·s), secretion rate constants for fats and carbohydrates *s*_(0)(2)(4)_ = 1.315 · 10^−7^/*V*_(2)_ kg/(m^3^·s), and *s*_(0)(2)(5)_ = 1.54 · 10^−7^/*V*_(2)_ kg/(m^3^·s). Similarly, we have calculated secretion rates constants for pepsin and sodium hydrogen carbonate in different tract portions. Ion exchange is almost instantaneous *k*_(0)(2)_ > 10^4^ m^3^/(kmol·s), and a minimum value is used in the calculations [60]. The kinetic constants of the enzyme reaction between pepsin and various complex proteins differ significantly [75]. When calculating, we used certain mean values of the reaction rate for synthetic protein C_20_H_22_N_2_O_4_ N-acetyl-L-phenylalanyl-L-phenylalanine (*M*_(3)_ = 354.4 g/mol) *k*_(3)(7)_ = 0.038/s, *k*_(3)(7)_′ = 1.4 · 10^−3^ kmol/m^3^ [61]. Constants *a*_(3)(7)_, *b*_(3)(7)_, *c*_(3)(7)_ are defined according to the data [62]. The maximum reaction rate is reached at pH = 2.32. When pH = 4.44 is exceeded, the reaction rate equals zero. We used lead to analyze absorption of chemicals from the GI tract into blood. The diffusion coefficient for lead is taken as 5.12 · 10^−10^ m^2^/s, and the permeability coefficient is taken as 3.34 · 10^−3^ s^−1^ [59]. The share of the lead component in a food particle is taken as *β*_(6)_ = 0.28 · 10^−6^ [76].

In our calculations, we used the parameters of a carrot for food particles since there is available experimental data on the dissolution rate as a function of pH level [64]. To approximate the mass-transfer coefficient *k*, the exponential function *k* = *k*_phys_ · *f*(pH) = *k*_phys_ · *a* · pH^*b*^, *a* = 3.246, *b* = −2.092 was used. The components content in raw carrots is assumed as equal to *β*_(3)_ = 0.013, *β*_(4)_ = 0.001, *β*_(9)_ = 0.893, and *β*_(5)_ = 0.093 − *β*_(6)_.

**Figure 3:**
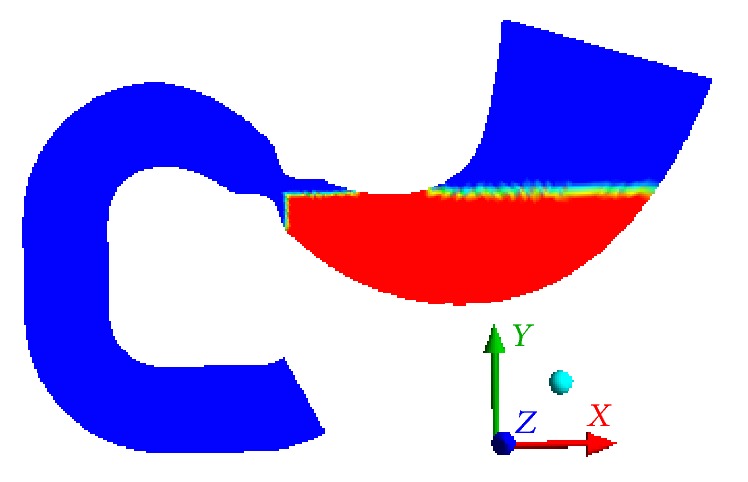
Initial filling with particles: blue indicates the first phase and red is the region filled with particles.

**Figure 4:**
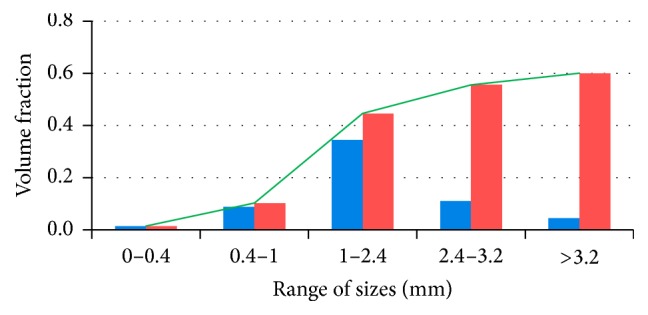
Initial mass fraction of the particles: blue bar displays volume fraction of particles in different range of sizes and red bar and green line show distribution function.

Parameters of the distribution function for the food particles after fragmentation [56] were set for carrots (20 chewing cycles, functionality of the dentition which equals 1) based on the available literature [77]. Due to high density, the initial layout of the food particles is set in bulge portion of stomach (Figure 3); volume fractions of phases are presented in Table 2. At a first approximation, we used five phases of food particles. Diagram of initial distribution function of particle sizes is presented in Figure 4. During food dissolution, some substances (proteins, fats, carbohydrates, chemicals, and water) are transitioned into a solution (first phase), and a particle becomes smaller. In other words, the mass is transitioned into the phase of particles with smaller size. The smallest particles cannot be dissolved (nondigestible fibers [58]). At the beginning, the first phase contains only a water component *Y*_(9)_^0^ = 1, *ρ*_(1)_ = 1000 kg/m^3^. Initial mass fractions of other components are equal to zero *Y*_(*i*)_^0^ = 0, $i=\bar{0,8}$. All initial carbohydrates/proteins/fats (*β*_(3)_, *β*_(4)_, *β*_(5)_) are contained in particles phases (Table 1). It is not physiological state. We have some basal concentrations of acid, alkaline, pepsin, and reaction product in reality. But we want to see how fast the spreading and saturating of secretory elements in the antroduodenum occurred.  Table 3 summarizes all component mass sources and initial mass fractions.

## 11. Results and Discussion

In Ansys Meshing, we built a computational grid that consists of 31317 prismatic elements. The length of surface elements edge varies from 2.7 · 10^−5^ m (in the areas of concave antrum portion and region near pyloric sphincter) to 5.5 · 10^−3^ m (in convex part of the antrum). Dynamic reconfiguration of the computational grid during the peristaltic activity is performed based on a script (user-defined function) written in *C*. The grid reconfiguration algorithm is based on the use of sinus quadratic function which defines the wave shape. It should be noted that the edge nodes can shift only in a respective elliptical cross-section, thus imitating contraction/relaxation of circular muscles.

In our studies, we analyzed several scenarios (Table 4). We also analyzed the impact of acid secretion disorder on food dissolution rate and the impact of alkaline secretion disorder on acidity level in the tract. Additionally, we looked into the relation between density and particle evacuation into the bowel.

According to the first basic scenario, the density of food particles equals *ρ*_(*j*)_ = 1040 kg/m^3^, $j=\bar{2,6}$, with no functional disorders in the tract *F*_(*l*)(*m*)_ = 1, $l=\bar{2,5}$, $m=\bar{1,3}$ (scenario 1).

In case of gastric disorders, the rate of gastric acid secretion can be significantly reduced, and it affects digestion negatively. For example, it is shown that, in patients with atrophic gastritis, the acid secretion rate is on average 40–50% lower than in healthy persons [78]. This reduction is observed for both the basal secretion and maximal secretion. In case of severe atrophic gastritis, the acid secretion rate can drop to several percent from the normal physiological level [79]. In scenarios 2 and 3, we analyze the results with acid secretion disorders at two levels: *F*_(2)(1)_ = 0.5 and *F*_(2)(1)_ = 0.2, which can correspond to atrophic gastritis of varying severity.

Reduction in the secretion of sodium bicarbonate can be caused by a number of disorders. For example, in patients with inactive duodenal ulcer, the basal bicarbonate secretion rate in duodenum is almost twice as low as that of healthy persons [80]. On the other hand, the rate of bicarbonate secretion from stimulation with hydrogen chloride in case of ulcer is almost three times lower. It is possible that reduced alkaline secretion resulted in deterioration of the mucous layer and ulcer. The secretion of sodium bicarbonate with pancreatic juice may also decrease due to functional disorders [81], age [82], or adverse factors, for example, smoking [83]. In scenarios 4 and 5, we analyzed the results assuming secretory function reduction of the antrum, duodenum, and pancreas altogether.

There are many current studies on the relation between the rate of gastric evacuation and the type (solid, liquid) [84], content (proteins, fats, and carbohydrates) [85], and osmolality of food [86]. Moreover, such physical parameters of the particles as density, solidity, and size also have their impact, but the shape of particles does not have a significant impact on the evacuation rate [36]. The use of a 3D tract model can improve our understanding of this issue. In the latter scenario, we consider the results of particle density *ρ*_(*j*)_ = 1005 kg/m^3^, $j=\bar{2,6}$ (scenario 6).

**Figure 5:**
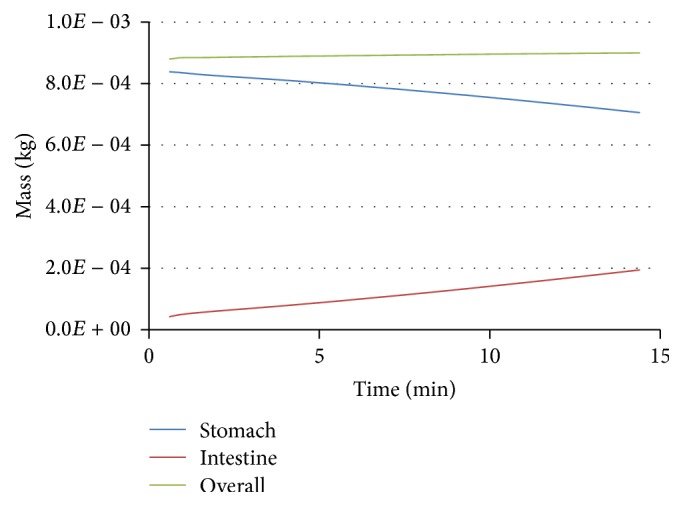
Mass change of a sixth phase of food particles in stomach and intestinal canal.

**Figure 6:**
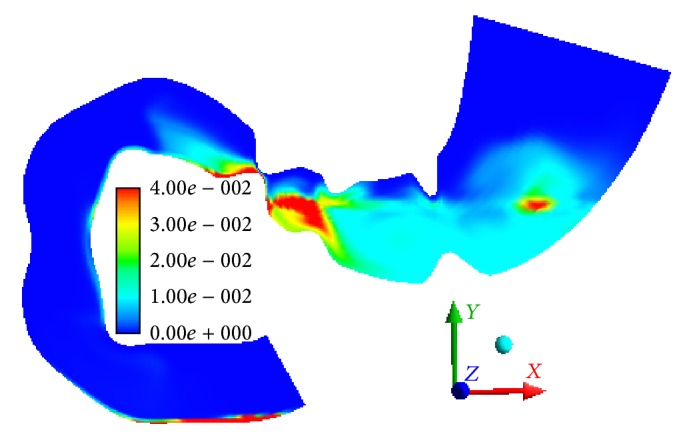
Volume fraction of particles of the sixth phase *α*_(6)_ (0.2 mm), *t* = 864 s.

The modeling results indicate that, in the first scenario, the particles sized >0.7 mm do not evacuate into the bowel but settle into the lower part of the stomach. Only the smallest particles (0.2 mm) can concentrate near the gastric outlet and can then transfer into the bowel at the opening of sphincter (Figures 5 and 6).

On the other hand, some studies have shown that the food particles sized < 2 mm are evacuated during fed motility [36]. This discrepancy might be caused by a significant impact of density on evacuation [87]. In scenarios 1–5, the difference of densities between the main liquid phase and the particle phases equals 40 kg/m^3^. In the course of digestion, this difference decreased due to mutual diffusion which is currently not reflected in the presented model. If the difference between the densities is lesser, it is possible that larger food particles might be evacuated into the bowel. This situation will be reviewed in scenario 6.

**Figure 7:**
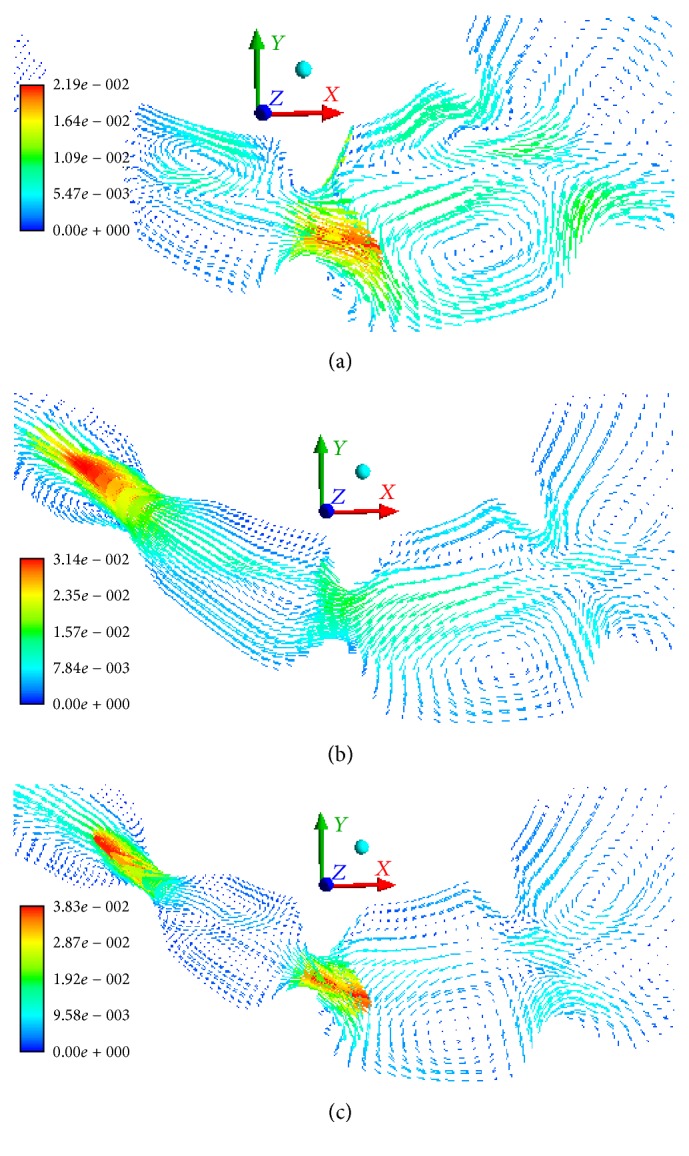
The velocity field of the sixth phase in the antrum and the sphincter zone, m/s (a) *t* = 855 s, (b) *t* = 858 s, and (c) *t* = 861 s.

**Figure 8:**
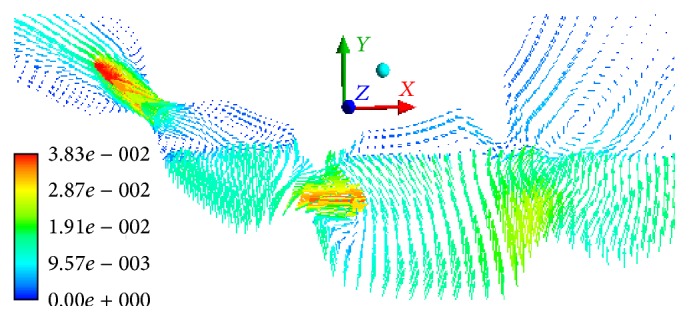
The velocity field of the first phase in the antrum and the sphincter zone, m/s, *t* = 861 s.

The mass of the smallest particles (phase 6) increases due to mass transition from phase 5. In 15 minutes, 20% of the sixth-phase particles are transitioned from the stomach into the bowel. When the gastric outlet is closing, we can observe the velocity of the sixth phase to 0.0219 m/s in the direction opposite to the wave propagation (Figure 7(a)). When the gastric outlet is opening, the particles transit into the bowel at 0.0314 m/s (Figure 7(b)). Regardless of the position of gastric outlet, we can observe circulating flows between the wave peaks in the antrum. At any point in time, we can observe the first-phase flow along axis *Y* in the lower portion of the stomach (in the area of particle accumulation) which is caused by its lower density as compared to the food particle phase (Figure 8).

When gastric outlet is closed, the velocity in the antrum is similar to the results obtained with the use of available models [43–47]. One of the first computer models of the stomach shows that the contraction wave in the antrum plays an important role [40]. The reverse flow of the liquid and circulating flows between the wave peaks promote active mixing of the gastric content. When the motor function is damaged (e.g., at a lesser wave amplitude), the flow velocity decreases, and the food mixing is not as effective [56]. When the contraction wave reaches the middle of the antrum, the gastric outlet opens, and the gastric content transfers into the bowel. One of the mechanisms that open the sphincter is the peristaltic waves that create increased pressure in the antrum. A damaged motor function slows down evacuation due to a lower flow rate.

**Figure 9:**
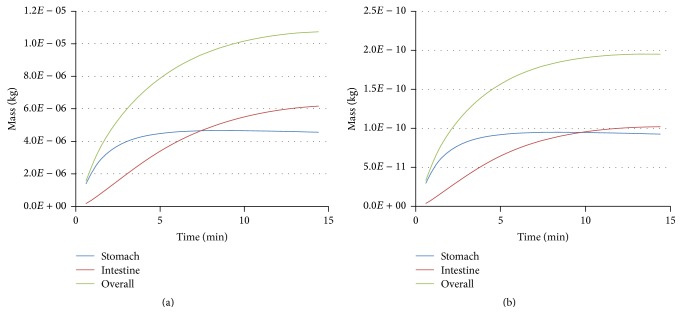
Change of the mass component of the dissolved protein (a) and lead (b) in stomach and intestinal canal.

**Figure 10:**
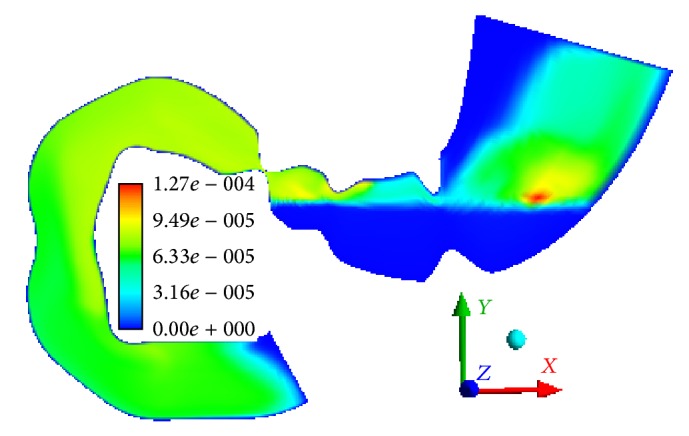
Mass fraction of dissolved proteins, *t* = 864 s.

**Figure 11:**
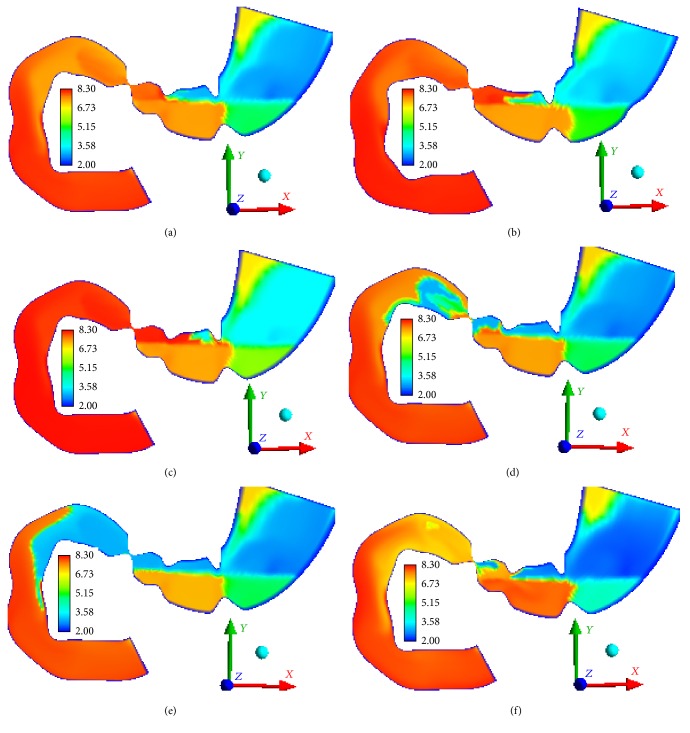
pH level *t* = 306 s, (a) scenario 1, (b) scenario 2, (c) scenario 3, (d) scenario 4, (e) scenario 5, and (f) scenario 6.

The ratio of the chemical substance masses (lead) in the stomach and the bowel differs from that of dissolved food components due to lead absorption via the tract wall (Figure 9). The mass of the dissolved substances is constantly growing due to the transition from the stomach into the bowel. The content of the first-phase components reaches a stationary level 5–15 min after the start of the modeled digestion stage (Figures 9 and 10). It is linked to the fact that the amount of food in the stomach does not change significantly over the period of time under consideration. The first-phase components spread rapidly over the modeled region due to advection (Figure 11). It is possible that when the motor function is damaged, advective mixing of the components will not be so active. In this case, the role of diffusion increases. It is practical to perform a further study of the impact that these effects have on digestion.

The total mass of the food particles (phases $j=\bar{2,6}$) decreases over time due to transition of the mass into the first-phase components. The mass reduction reaches its peak in the phases of a smaller size ($j=\bar{4,5}$) which is connected to a larger specific surface area (Table 5).

The mass change rate of the *j* = 2 phase particles is higher than the mass change rate of the *j* = 3 phase particles. This happens because the mass of the third-phase particles increases due to dissolution of the second-phase particles. At the same time, the mass of the second-phase particles does not increase at the expense of smaller particles. Smallest particles (phase *j* = 6) cannot be dissolved. Their total mass increases due to the mass transition from larger particles (from phase *j* = 5). When hydrogen chloride secretion is damaged (scenario 3), the particles dissolve at a slower rate because in this case the acidity level in stomach is significantly lower.

In the first scenario, the pH level in the intestinal canal changes from 7.3 to 8.3 that is consistent with alkaline environment; the environment in the upper portion of stomach has a high level of acidity; the pH level reaches 2-3; in the area of the gastric outlet, the pH level goes up to 8 which is caused by neutralization of acid by sodium hydrocarbonate secreted in this area (Figure 11(a)). When acid secretion is damaged, the pH level in the upper portion of stomach increases to 4-5 depending on the severity of damage (Figures 11(b) and 11(c)). The rate of particle dissolution drops dramatically (scenario 3 in Table 5).

The obtained results correspond to the available experimental data: in patients with atrophic gastritis, an average gastric pH is about 5.1; in healthy persons, the value stands at 2.7 [88]. When hydrocarbonate secretion is damaged, the pH level in the upper portion of the duodenum reduces to 3-4 (Figures 11(d) and 11(e)), and it can cause damage to the mucous layer of the tract. These results are confirmed by the available literature: patients with duodenal ulcer disease have longer periods of increased acidity in the duodenum (pH < 4) [89].

Let us briefly discuss the results obtained for the food particles of lesser density (scenario 6). The results of computational modeling indicate that in this case the particles sized smaller than 0.7 mm (phases *j* = 5 and *j* = 6) enter the bowel. Besides, the gastric content mixing is enhanced (the rate reaches 0.05 m/s). We can also observe increased acidity (Figure 11(f)) and faster dissolution of the food particles.

It is possible that the particle density is a significant factor that affects gastric evacuation. In the beginning of the digestion process, dense particles settle at the bottom of the stomach. Due to the diffusion of gastric juice into food particles, the difference between the densities of the main phase and the particles decreases. Evacuation of larger particles begins; they have been soaked in the juice and are ready to be digested in the following tract portions. Consequently, the use of mathematical modeling gives a whole new insight into the process of food decomposition and homogenization.

## 12. Conclusions and Future Research

To conclude, the suggested model describes the multiphase flow in antroduodenal portion of gastrointestinal tract under normal conditions and in case of pathologic disorders. We should note that the predictive value of the model will improve with the development of other systems' submodels. The described model has two main advantages; firstly, it includes all the digestive system functions, namely, secretory, motor, and absorption. Secondly, it considers reactions and process of food particles dissolution. Currently, the nervous system regulation process has been indirectly covered in the study through feedback in the secretory elements. We will require further studies to analyze the impact of nervous system on the tract motor function. Also, we are planning to include enteric enzymes into the model and examine respective reactions for conjugated fats, proteins, and carbohydrates decomposition.

The paper does not focus on disorder accumulation mechanisms at the “mesolevel,” such as the direct damage that chemical substances and secreted hydrogen chloride do to the tract walls depending on distribution of their concentrations in the tract. We will probably need to further divide the gastric regions adjacent to the bulge and inverted portions as well the gastric outlet and duodenum regions. Future studies can focus on temperature effects including the damage of the tract walls caused by cold or hot food. When analyzing the hydrocarbonate secretion in the near-wall layer, we should remember that in reality the tract wall is covered by a layer of mucous. This layer has specific properties and protects the tract walls from damage. The mucous layer is only ≈0.001 m (microscale) thick, but the difference in pH levels at the layer border can reach 5 [90]. This difference corresponds to the neutral environment near the wall pH = 7 and acid environment in the stomach pH = 2. Further analysis performed at two levels (flows in the stomach and processes in the mucous level) is also an interesting subject of future research.

In the discussion of the mucous layer and the mechanisms behind deterioration of the tract wall, we bear* Helicobacter pylori* bacteria in mind. Their major role in the development of gastritis, gastric ulcer, and duodenal ulcer is proved [91]. Several approaches have been already developed that describe bacteria migration in the mucous layer and tract examining their impact on the acid secretion process [34]. Development of a 3D migration model is also an interesting research task.

Ulcers start mostly in the antroduodenum. And here the suggested model can predict high risk regions. The regions may vary due to the individual characteristics of a tract shape. In the future, it might be interesting to analyze the influence that individual parameters of a shape have on evacuation of food particles into the bowel. The obtained characteristics of evacuation can be used in optimization of drug delivery to the bowel. One more way to develop the model is introducing a free “air-liquid” surface for a more comprehensive analysis of food evacuation process.

Further identification of the model parameters is still an important aspect of study: for example, in the general case the coefficient of mass transition from a particle into the solution *k* should depend on the forces affecting a particle as well as on physicochemical and physical-mechanical properties of a particle. Additionally, it is necessary to conduct an experimental study to determine rheological characteristics of the gastric content since viscosity and density of the environment are not constant, and they change in the digestion process [92–94].

The challenges that we have faced in our study include the lack of experimental data for the identification of more comprehensive models. Moreover, the identification process is exacerbated by the fact that many of the model parameters may differ from individual to individual. The parameters have to be set for an individual patient. And finally, further improvement of the model requires higher computer performance with using parallel calculations and multiprocessing systems.

## Figures and Tables

**Table 1:** Parameters values of the model.

Parameter	Value	Source
Secretion/absorption		
*s*_(0)(2)(3)_	~1.616 · 10^−7^/*V*_(2)_ kg/(m^3^·s)	Data [58]
*s*_(0)(2)(4)_	~1.315 · 10^−7^/*V*_(2)_ kg/(m^3^·s)	Data [58]
*s*_(0)(2)(5)_	~1.54 · 10^−7^/*V*_(2)_ kg/(m^3^·s)	Data [58]
*s*_(0)(2)_^0^	~0.404 · 10^−7^/*V*_(2)_ kg/(m^3^·s)	Estimation by [58]
*s*_(0)(2)_′	~12.66 kg/m^3^	Estimation by [58]
*s*_(9)(2)_	~171.3	Estimation by [58]
*s*_(7)(2)_^0^	~0.416 · 10^−8^/*V*_(2)_ kg/(m^3^·s)	Data [58]
*s*_(7)(2)_	~1.664 · 10^−8^/*V*_(2)_ kg/(m^3^·s)	Data [58]
*s*_(7)(2)_′	~12.66 kg/m^3^	Estimation by [58]
*s*_(6)(2)_	3.34 · 10^−3^ s^−1^	[59]
*s*_(6)(3)_	3.34 · 10^−3^ s^−1^	[59]
*s*_(6)(4)_	3.34 · 10^−3^ s^−1^	[59]
*s*_(6)(5)_	3.34 · 10^−3^ s^−1^	[59]
*s*_(2)(4)_^0^	~0.278 · 10^−8^/*V*_(4)_ kg/(m^3^·s)	Data [58]
*s*_(2)(4)_	~1.112 · 10^−8^/*V*_(4)_ kg/(m^3^·s)	Data [58]
*s*_(2)(4)_′	~0.5 kg/m^3^	Estimation by [58]
*s*_(9)(4)_	~395.5	Estimation by [58]
*s*_(2)(5)_^0^	~1.972 · 10^−7^/*V*_(4)_ kg/(m^3^·s)	Data [58]
*s*_(2)(5)_	~7.888 · 10^−7^/*V*_(4)_ kg/(m^3^·s)	Data [58]
*s*_(2)(5)_′	~0.5 kg/m^3^	Estimation by [58]
*s*_(9)(3)_	~395.5	Estimation by [58]
*s*_(9)(5)_	~78.3	Estimation by [58]
*s*_(2)(3)_^0^	~1.75 · 10^−8^/*V*_(3)_ kg/(m^3^·s)	Data [58]
*s*_(2)(3)_	~7 · 10^−8^/*V*_(3)_ kg/(m^3^·s)	Data [58]
*s*_(2)(3)_′	~0.5 kg/m^3^	Estimation by [58]

Reactions, solution of particles		
*K*_*i*_	1 · 10^−9^ m^2^/s	Max. [60]
*k*_(0)(2)_	10^4^ m^3^/(kmol·s)	Min. [60]
*k*_(3)(7)_	0.038/s	[61]
*k*_(3)(7)_′	1.4 · 10^−3^ kmol/m^3^	[61]
*c*_(3)(7)_	−4.857	Estimation by [62]
*b*_(3)(7)_	26.819	Estimation by [62]
*a*_(3)(7)_	−5.786	Estimation by [62]
*M*_(3)_	354.4 g/mol	Scenario
*M*_(7)_	35000 g/mol	Scenario

Scenario (carrot)		
β_(6)_	0.28 · 10^−6^	[63]
β_(3)_	0.013	Scenarios
β_(4)_	0.001	Scenarios
β_(5)_	0.093 − β_(6)_	Scenarios
β_(9)_	0.893	Scenarios
*a*	3.246	Estimation by [64]
*b*	−2.092	Estimation by [64]
*k*_phys_	2.72 · 10^−4^ kg/(m^2^·s)	Estimation by [64]
ρ_(*j*)_, $j=\bar{2,6}$	1040 kg/m^3^	Scenarios 1–5

**Table 2:** Initial volume fraction of the particles in the region filled with particles.

Phase	Range of sizes, mm	Medium size, mm	Fraction, α_(*j*)_^0^
*j* = 1	—	—	0.4
*j* = 2	>3.2	3.6	0.044
*j* = 3	2.4–3.2	2.8	0.110
*j* = 4	1–2.4	1.7	0.344
*j* = 5	0.4–1	0.7	0.088
*j* = 6	0–0.4	0.2	0.014

**Table 3:** Component mass sources and initial mass fractions.

Component index	Component name	Initial fraction, *Y*_(*i*)_^0^	Secretion sources	Absorption sources	Reaction sources	Source from particles
*i* = 0	Hydrochloride acid	0	*S*_(0)(2)_	—	−*R*_(0)_	—
*i* = 1	Reaction product	0	—	—	*R*_(1)_	—
*i* = 2	Sodium bicarbonate	0	*S*_(2)(*l*)_, *l* = 3,4, 5	—	−*R*_(2)_	—
*i* = 3	Solute proteins	0	—	—	−*R*_(3)_	∑_*j*_ *m*_(*j*)(3)_′
*i* = 4	Solute fats	0	—	—	—	∑_*j*_ *m*_(*j*)(4)_′
*i* = 5	Solute carbohydrates	0	—	—	—	∑_*j*_ *m*_(*j*)(5)_′
*i* = 6	Chemicals	0	—	−*S*_(*i*)(*l*)_, $l=\bar{2,5}$	—	∑_*j*_ *m*_(*j*)(6)_′
*i* = 7	Pepsin	0	*S*_(7)(2)_	—	—	—
*i* = 8	Polypeptides	0	—	—	*R*_(8)_	—
*i* = 9	Water	1	*S*_(9)(*l*)_, $l=\bar{2,5}$	—	*R*_(9)_	∑_*j*_ *m*_(*j*)(9)_′

**Table 4:** Model scenarios.

Number	Functional acid secretion disorders, *F*_(2)(1)_	Functional alkaline secretion disorders, *F*_(*l*)(1)_, $l=\bar{3,5}$	ρ_(*j*)_, $j=\bar{2,6}$, kg/m^3^
1	1	1	1040
2	0.5	1	1040
3	0.2	1	1040
4	1	0.5	1040
5	1	0.2	1040
6	1	1	1005

**Table 5:** Changes in the food particle mass over 306 s.

Phase	Initial mass, kg	Change of the mass, scenario 1	Change of the mass, scenario 3	Change of the mass, scenario 5
*j* = 2	2.84*E* − 03	−2.09%	−1.68%	−2.19%
*j* = 3	7.09*E* − 03	−1.45%	−1.17%	−1.52%
*j* = 4	2.21*E* − 02	−2.30%	−1.80%	−2.43%
*j* = 5	5.60*E* − 03	−4.73%	−3.47%	−5.13%
*j* = 6	8.80*E* − 04	1.12%	0.96%	1.18%
*j* = 2–6	3.85*E* − 02	−2.40%	−1.86%	−2.56%

## Symbols

*a*, *b*: Nondimensional coefficients of power dependence on pH for the rate of mass transfer from particle into the first-phase, *a* > 0, *b* < 0
*a*_(3)(7)_, *b*_(3)(7)_, *c*_(3)(7)_: Nondimensional coefficients of quadratic dependence on pH for the enzymatic reaction rate
*C*_(*i*)_^*b*^: Mass concentration of an *i*-chemical in blood, kg/m^3^
*C*_(*i*)_: Mole concentration of the first-phase *i*-component, kmol/m^3^, $i=\bar{0,I}$
*d*_(*j*)_: A particle diameter of a *j*-phase, $j=\bar{2,n}$
*D*_(*l*)(*m*)_ ∈ [0,1]: Damage in the *l*-sub-area of the antroduodenal part of the digestive tract, characterizing *m*-function (*m* = 1,2, 3 stand for secretion, absorption, and motor activity, resp.), $l=\bar{2,5}$
*F*_(*l*)(*m*)_: The functionality of the *l*-sub-area, characterizing *m*-function $l=\bar{2,5}$
*h*_(*i*)_: A nondimensional coefficient showing the critical concentration level of an *i*-substance in tract cavity and blood, at which the diffusion into blood starts
$i=\bar{0,I}$: The first-phase component index: hydrochloride acid (*i* = 0), reaction product of hydrochloride acid and sodium hydrogen carbonate (carbon dioxide and sodium chloride) (*i* = 1), sodium hydrogen carbonate (*i* = 2), solute proteins (*i* = 3), fats (*i* = 4), carbohydrates (*i* = 5), chemicals (*i* = 6), pepsin (*i* = 7), polypeptides (*i* = 8), and water (*i* = 9)
$j=\bar{1,n}$: The phase index; index *j* = 1 corresponds to the first phase, multicomponent liquid, and index *j* > 1 corresponds to phases representing food particles of different sizes, which in first approximation are being described with a model of liquid with different viscosity
*k*_(0)(2)_, *k*_(3)(7)_, *k*_(3)(7)_′: Reactions rates constants, m^3^/(s·kmol), 1/s, kmol/m^3^
*k*: A mass-transfer coefficient from a particle into the first phase, kg/(m^2^·s), (mass transition speed from a unit of area); it is given with the power function of pH: *k* = *k*_phys_ · *f*(pH) = *k*_phys_ · *a* · pH^*b*^, where *k*_phys_ is the mass-transfer coefficient from a particle into the first phase at physiologically normal level of acid (*f*(pH) = 1) in the body of the stomach, kg/(m^2^·s)
*K*_(*j*)(*q*)_: A coefficient of interphase interaction *j* and *q*-phase, kg/(m^3^·s), $j=\bar{1,n}$, $q=\bar{1,n}$
*K*_(*i*)_: A diffusion coefficient for the first-phase *i*-component in digestive tract cavity, m^2^/s, $i=\bar{0,I}$
*m*_(*j*)(*i*)_′: An augend in the mass balance equation, determining the transition intensity from a *j*-phase into the first-phase *i*-component, kg/(m^3^·s), $i=\bar{0,I}$, $j=\bar{2,n}$
*m*_(*j*−1)(*j*)_′′: Addend in the mass balance equation, determining the transition intensity from a (*j* − 1)-phase into a *j*-phase, kg/(m^3^·s), $j=\bar{2,n}$, *m*_(1)(2)_′′ = *m*_(*n*)(*n*+1)_′′ = 0
*M*_(*i*)_: The molar mass of the first-phase *i*-component, kg/kmol, $i=\bar{0,I}$
*p*: The phases mixture pressure, Pa
pH: The medium acidity level
*R*_(*i*)_: The mass source intensity of the first-phase *i*-component due to reactions between components, kg/(m^3^·s), $i=\bar{0,I}$
*S*_(*i*)(*l*)_: The intensity of mass sources of the first-phase *i*-component in the area *Ω*_(*l*)_, kg/(m^3^·s), $i=\bar{0,I}$, $l=\bar{1,L}$
*s*_(*i*)(*l*)_^0^, *s*_(*i*)(*l*)_′, *s*_(*i*)(*l*)_: The secretion/absorption rates constants for the first-phase *i*-component in the area *Ω*_(*l*)_, kg/(m^3^·s), kg/m^3^, kg/(m^3^·s), $i=\bar{0,I}$, $l=\bar{1,L}$
*t*: The independent variable (time), s
*V*_(*l*)_: The volume of the area *Ω*_(*l*)_, m^3^
*Y*_(*i*)_: The mass fraction of the first-phase *i*-component, ∑_*i*_ *Y*_(*i*)_ = 1, $i=\bar{0,I}$,
*α*_(*j*)_: The volume fraction of the *j*-phase, ∑_*j*_ *α*_(*j*)_ = 1, $j=\bar{1,n}$,
*β*_(*i*)_: The mass fraction of the *i*-component in food particles, ∑_*i*_ *β*_(*i*)_ = 1: food particles consist of proteins, fats, carbohydrates, chemicals, and water, respectively, *i* = 3,4, 5,6, 9
*η*_(*j*)_: The shear viscosity of the *j*-phase, Pa·s, $j=\bar{1,n}$
*ρ*_(*j*)_: The density of the *j*-phase, kg/m^3^, $j=\bar{1,n}$
[*ρ*_(1)_*α*_(1)_*Y*_(*i*)_]_(*l*)_: The mean mass concentration of the first-phase *i*-component in the area *Ω*_(*l*)_, kg/m^3^, $i=\bar{0,I}$, $l=\bar{1,L}$
*Ω*: The whole area interior; ∂*Ω*, the area boundary; $\bar{\Omega }=\Omega \cup \partial \Omega$, closed area (area interior and its boundary); ∂*Ω*_(*l*)_, *l*-boundary of the area, $l=\bar{1,L}$; *Ω*_(*l*)_, internal layer, adjacent to the *l*-boundary, $l=\bar{1,L}$
**g**: A vector, characterizing bulk forces impact, m/s^2^
**J**_(*i*)_: A mass flow intensity vector of the first-phase *i*-component due to diffusion processes, kg/(m^2^·s), $i=\bar{0,I}$
**r**: A radius-vector of spatial points, m, **r** = *x* **e**_*x*_ + *y* **e**_*y*_ + *z* **e**_*z*_ = {**x**, **y**, **z**} in Cartesian coordinate system
**v**_(*j*)_: A vector of *j*-phase velocity, m/s, $j=\bar{1,n}$
**τ**_(*j*)_: A stress tensor of the *j*-phase, Pa, $j=\bar{1,n}.$
